# Carboxylated graphene oxide nanosheets as efficient electrodes for high-performance supercapacitors

**DOI:** 10.3389/fchem.2022.944793

**Published:** 2022-08-29

**Authors:** Hassan Idris Abdu, Hamouda Adam Hamouda, Joshua Iseoluwa Orege, Mohammed Hassan Ibrahim, Anas Ramadan, Taslim Aboudou, Hongxia Zhang, Jinjin Pei

**Affiliations:** ^1^ Qinba State Key Laboratory of Biological Resources and Ecological Environment, 2011 QinLing-Bashan Mountains Bioresources Comprehensive Development C. I. C, Shaanxi Province Key Laboratory of Bio-resources, College of Bioscience and Bioengineering, Shaanxi University of Technology, Hanzhong, China; ^2^ College of Chemistry and Chemical Engineering, Northwest Normal University, Lanzhou, China; ^3^ Department of Chemistry, Faculty of Science, University of Kordofan, El Obeid, Sudan; ^4^ Ekiti State University, Ado-Ekiti, Nigeria; ^5^ University of Chinese Academy of Sciences, Beijing, China; ^6^ The First Hospital of Lanzhou University, Lanzhou University, Lanzhou, China

**Keywords:** supercapacitor, carboxylated graphitic nanosheets, ball-milling, high performance, electrodes, specific capacitance

## Abstract

In the presence of dry ice, a series of graphitic materials with carboxylated edges (ECGs) were synthesized by ball milling graphite for varied times (24, 36, and 46 h). The influence of carboxylation on the physiochemical characteristics and electrochemical performance as effective electrodes for supercapacitors were assessed and compared with pure graphite. Several characterization techniques were employed to investigate into the morphology, texture, microstructure, and modification of the materials. Due to its interconnected micro-mesoporous carbon network, which is vital for fast charge-discharge at high current densities, storing static charges, facilitating electrolyte transport and diffusion, and having excellent rate performance, the ECG-46 electrode among the investigated samples achieved the highest specific capacitance of 223 F g^−1^ at 0.25 A g^−1^ current density and an outstanding cycle stability, with capacitance retention of 90.8% for up to 10,000 cycles. Furthermore, the symmetric supercapacitor device based on the ECG-46 showed a high energy density of 19.20 W h kg^−1^ at 450.00 W kg^−1^ power density. With these unique features, ball milling of graphitic material in dry ice represents a promising approach to realize porous graphitic material with oxygen functionalities as active electrodes.

## Graphical Abstract

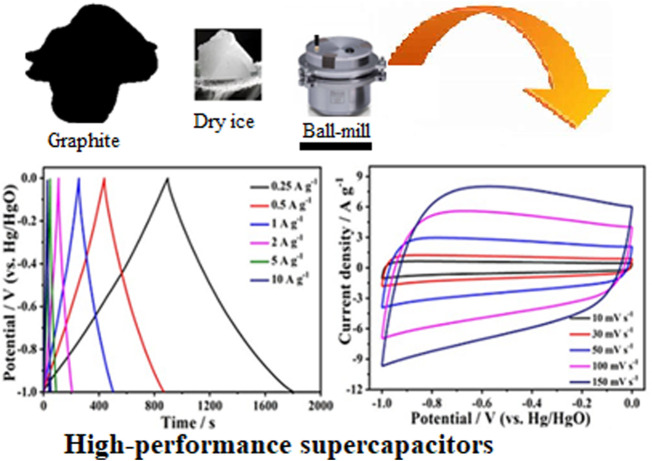

## Highlights

❖ Carboxylated-edge graphitic (ECG) nanosheets was efficient as electrodes for supercapacitor
❖ The ECG-46 electrode demonstrated excellent electrochemical performance
❖ The high performance of ECG-46 is due to its interconnected micro-mesoporous carbon network
❖ The symmetric supercapacitor device based on ECG-46 showed a high energy density

## Introduction

Supercapacitors have a long history in the field of energy storage (Conway and Pell, 2003; Rehman et al., 2022). Since Becker’s 1957 patent on a carbon electrode supercapacitor with an energy density comparable to batteries and a specific capacitance 3–4 orders of magnitude larger than conventional capacitors (Lu et al., 2014), great deals of efforts have been committed to the exploration and exploitation of carbonaceous resources (Wang et al., 2017; Ren et al., 2022). Carbon-based electrodes are notorious with their outstanding thermal-chemical stability, great electrical conductivity, and rich electron density, which allowed their utilization in various applications rooting from catalysis to energy production and conversion technologies (Bora et al., 2021; Eid et al., 2019a; Eid et al., 2019b; Eid et al., 2019c; Eid et al., 2019d; Xu X. et al., 2020; Liu et al., 2021; Q. Lu et al., 2021; Rasal et al., 2021). Additionally, they are affordable, widely accessible, and environmentally beneficial (Abdu et al., 2020a; Abdu et al., 2020b; Eid et al., 2022). Today, over 80% of supercapacitor products commercially available in the market are made of carbon-based nanostructural materials (Hao et al., 2013; Béguin et al., 2014). To maximize the performance properties of carbon material-based electrodes, integration of oxygen functionalities have been regarded as a promising surface enhancement strategy (Lin et al., 2011). Several papers have reported that such modification can improve their accessibility to electrolyte, capacitance and electrochemical activities.

Among the various forms of carbon-based materials, graphitic materials (such as graphene oxides) are considered potential electrodes for super capacitors because of their high flexibility and tunable properties, high electrical conductivity and light weight (Das et al., 2017; Guo et al., 2020). They are quite popular as energy storage materials because of their distinctive structure and capacity to introduce different functionality to their surfaces. Modification of graphitic materials through integration of oxygen-containing functions by oxidation-exfoliation-reduction could couple as much as possible oxygen-based pseudo-capacitance to graphene-based super-capacitor maintaining their long cycle-life and great power density (Bagley et al., 2021). It has been reported previously that acid-assisted functionalization incorporate sufficient amount of oxygen-containing functions for enhanced capacitor performance (Fang et al., 2012). Although graphene oxide has a low concentration of carboxylic groups at its edges, modification with a high concentration of carboxylic acid functions through carboxylation would boost its loading efficiency and widen their range of performance features (Chauhan et al., 2016; Vacchi et al., 2016). The performance of graphitic materials as the active electrode for high-performance supercapacitors must therefore be controlled through controlled loading of carboxyl moieties. In this study, we present a simple method for producing edge-carboxylated graphitic (ECG) nanosheets by ball milling graphite with dry ice. The relationship between the structure-electrochemical performances of the ECG nanosheets was uncovered. The ECGs exhibited remarkable virtues of interconnected micro-mesoporous carbon network, good conductivity and excellent electrochemical performance properties with a high energy density of 19.20 W h kg^−1^ at power density of 450.00 W kg^−1^.

## Experimental section

### Preparation of edge-carboxylate graphitic materials

The series of edge-carboxylated graphitic (ECG) nanosheets were prepared by grinding graphite with a planetary ball-mill machine as previously reported (Jeon et al., 2012; Idris Abdu et al., 2020) with slight modification over a period of 24, 36, and 46 h with dry ice (Alfa, GmbH and Co. KG, Beijing, China). Typically, 3 g powder graphite (Alfa-aesar, 100 mesh) and 15 g dry ice were sequentially loaded into capsules (made of stainless steel), containing twenty 2.5 mm diameter stainless steel balls and were rapidly closed before fixing in a planetary ball mill (PM 200). Then, agitation was performed at 550 revolutions per minute for 24, 36, and 46 h, respectively and internal pressure of the capsules was maintained at 3.0 bars. Finally, the capsule was unsealed in open air and a vast flash caused by carboxylated graphite particles appeared. The resultant product was Soxhlet extracted with (1 M) HCl. The absence of residual HCl in the as-prepared ECGs nanosheets was validated using silver nitrate indicator. The resultant edge-carboxylated graphitic (ECG) nanosheets prepared in 24, 36 and 46 h were designated as ECG-24, ECG-36 and ECG-46 respectively.

### Characterization

Scanning electron microscope (Tokyo, Japan, S-4800 Hitachi) and energy dispersive spectrometer (EDS) as well as transmission electron microscopy (Japan, JEM-1200EX) were used to characterize the morphological features of ECG materials. Chemical state of the material was determined using X-ray photoelectron spectroscopy (Germany, Escalab 210 system). X-ray diffraction patterns were detected using X Pert-Pro MPD, PANalytical Co. equipment (Almelo, Netherlands). Raman spectrometer fitted with an Argon ion laser (ʎ = 514.5 nm) was used to Raman spectra at room temperature. Before measuring nitrogen adsorption, the micromeritics of nitrogen adsorption (ASAP 2020) method was employed to evaluate the pore architectures of carbon samples. Prior to the sorption tests, all samples were degassed at 200°C.

### Electrochemical analysis

The electrochemical analysis of the electrode materials were performed in a three electrode system comprising the graphitic (1 *×* 1 cm^2^) sample (active material), platinum wire (counter electrode) and Hg/HgO (reference electrode), all of which are immersed in a 2 M KOH solution at ambient temperature in a CHI 660D electrochemical workstation. The working electrode was made by slurring 8:1:1 mass ratio of ECG sample, carbon black, and polyvinylidene fluoride (PVDF in a drop of N-methyl pyrrolidinone solution. Nickel foam was employed as current collector. After drying at 60°C for 24 h, the Ni foam (1.0 cm^2^) was weighed and pressed into sheets under 15 MPa. Each electrode’s total mass was limited to between 3.0 and 5.0 mg. Cyclic voltammetry (CV), Galvanostatic charge-discharge (GCD) and electrical impedance spectroscopy (EIS) measurements were tested. The cycle-life was also studied. The three-electrode cell was tested in a 2 M aq. KOH electrolyte. Similarly, in the potential window of the CV and GCD studies, various scan rates (10–150 mV s^−1^) and current densities (0.25–10 A g^−1^) were utilized (−1–0 V). The as-prepared working electrodes were computed for specific capacitance using the Eq. 1.

$$C_{m}=I\Delta t/\left(m\Delta V\right)$$ (1)

Where, I = discharge current (A), Δt = time of discharge (s), m = weight of active material (g), and ΔV = potential window (V), C_m_ = specific capacitances (F g^−1^) for the three-electrode system.

## Results and discussion

### Physicochemical properties of the graphitic materials

**FIGURE 1:**
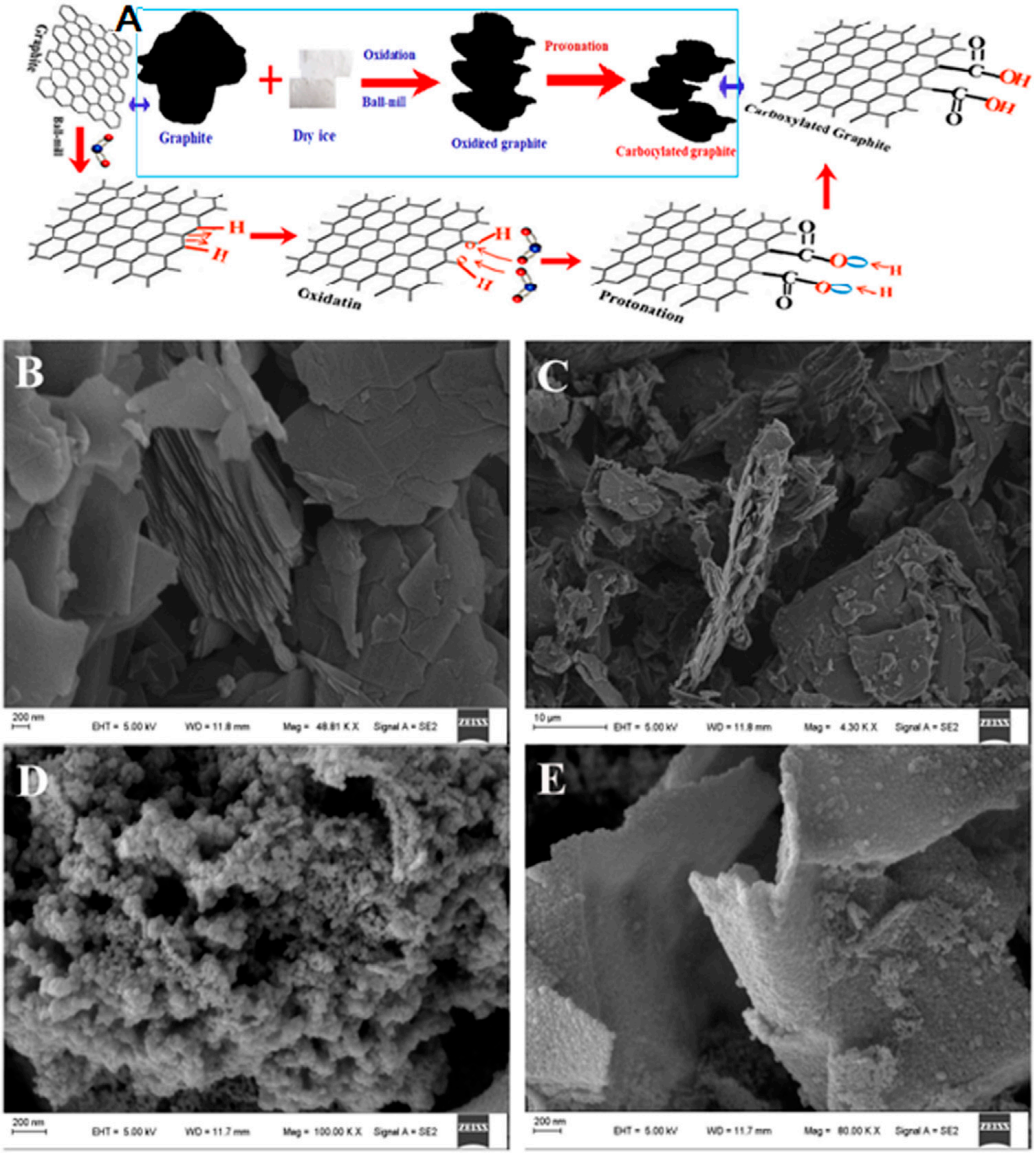
**(A)** Fabrication process and proposed mechanism schematic depiction; SEM image: **(B)** pristine graphite **(C)** ECG-24 **(D)** ECG-36 and **(E)** ECG-46.

Figure 1A depicts a schematic illustration of a mechanochemical process of graphite milling in the presence of dry ice for the preparation of carboxylated-edge graphitic materials. Physical crushing is enabled *via* ball milling, which decreases the graphite grain size, resulting in significant edge distortion and allowing for dry ice oxidation. The carbonyl group (COO^−^) becomes acidic when exposed to HCl, leading in an ECG system with a particularly rich COOH on edge. The SEM images show the typical morphology of graphite, ECG-24, ECG-36, and ECG-46 samples Figures 1B–E. In the figures, the as-prepared materials have different architectures. Before adding dry ice, the typical morphology of isolated 2D nanosheets with tandem layers at the surface can be seen in the pristine graphite (Figure 1B). Graphite nanosheets have lateral diameters of tens of micrometers, and it is not like ECGs, which have a lot of pores between isolated 2D nanosheets. The morphology of ECG-24 seen in Figure 1C showed a three-dimensional interconnected structure made up of numerous porous nanosheets woven together. However, as seen in Figure 1D, ECG-36 possessed a bulk shape with a very substantial hollow on the surface. In comparison with the materials above, the ECG-46 appears to exhibit most pores with no specific structure connecting adjacent pores, indicating that the 46-h milling period is suitable for creating an interconnected porous carbon network. Furthermore, as shown in Figure 1E, ECG-46 has a strongly linked pore structure with multiple macrospores and mesopores that allow for rapid ion transport, ion buffering, and ion storage, ensuring that the porous surfaces are well utilized (Chen et al., 2017; Gopalakrishnan and Badhulika, 2020; Wei et al., 2020) The micro-mesopore structure is particularly effective for rapid charge/discharge at high current densities and exceptional rate capability performance because multiple micropores may offer large specific surface areas for storing static charges (Zhang et al., 2018) (Oschatz et al., 2014). Interconnected mesopores may facilitate electrolyte transport and diffusion at the same time. (Bath et al., 2000; Li et al., 2016; Rybarczyk et al., 2016). The porous carbon structure would generate a one-of-a-kind open-pore system with a short electrolyte ion diffusion channel, resulting in increased electrochemical performance (Fan et al., 2012; Gui et al., 2013).

**FIGURE 2:**
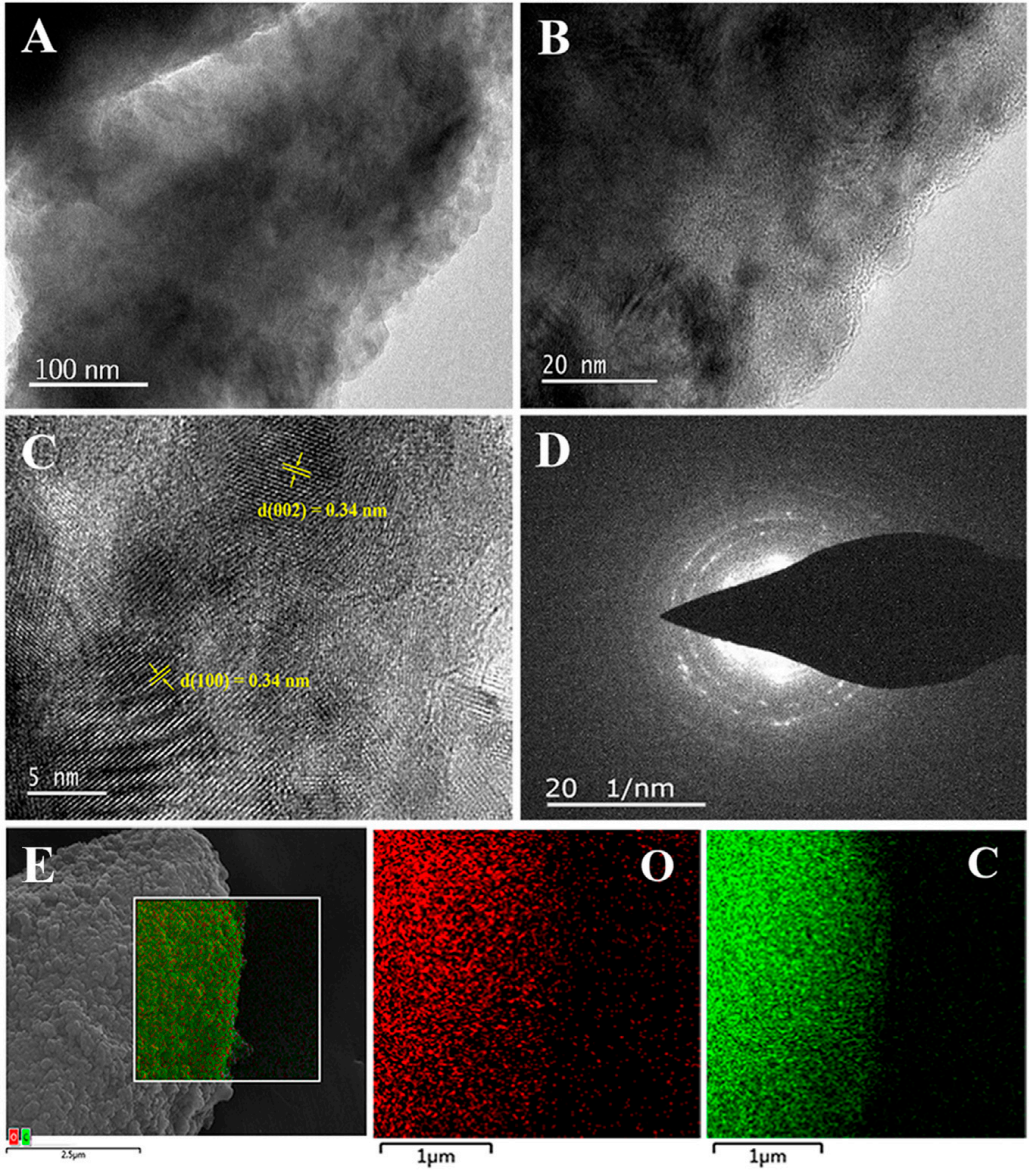
**(A,B)** TEM images of ECG-46 nanosheets at different magnification; **(C)** HRTEM image; **(D)** SAED pattern; and **(E)** dark-field SEM image and the elemental mapping of oxygen (O) and carbon (C) of the ECG-46 nanosheets.

The structure of the ECG-46 was confirmed using a TEM image (Figure 2). ECG-46 exhibits a substantial area of a thin, plate-like structure, as observed in low-magnification TEM images (Figure 2A). The carbon particles in ECG-46 have a porous structure that covers their inner surface. It completely covers the outside surface, showing that the porosity was effectively produced during the milling of graphite in the presence of dry ice. HRTEM images of the carbon nanosheet edges (Figure 2B) revealed ECG-46 layer structures with structural defects and network disorder, which are essential in the electrolyte ion and charge adaption areas. The HR-TEM image of the ECG-46 revealed a series of distinct retinal endpoints, each with an interplanetary distance of 0.34 nm in both the (002) and (100) planes, as shown in Figure 2C. The material was further examined using a selected area electron diffraction (SAED) test. The well-defined diffraction rings in Figure 2D indicate that ECG-46 is an amorphous carbon structure with a large number of holes. The diffraction rings of ECG-46 can be indexed to the carbon phase levels 002 and 100. SEM image element mapping analysis revealed that the two atoms of C and O were evenly distributed in the ECG-46 (Figure 2E).

**FIGURE 3:**
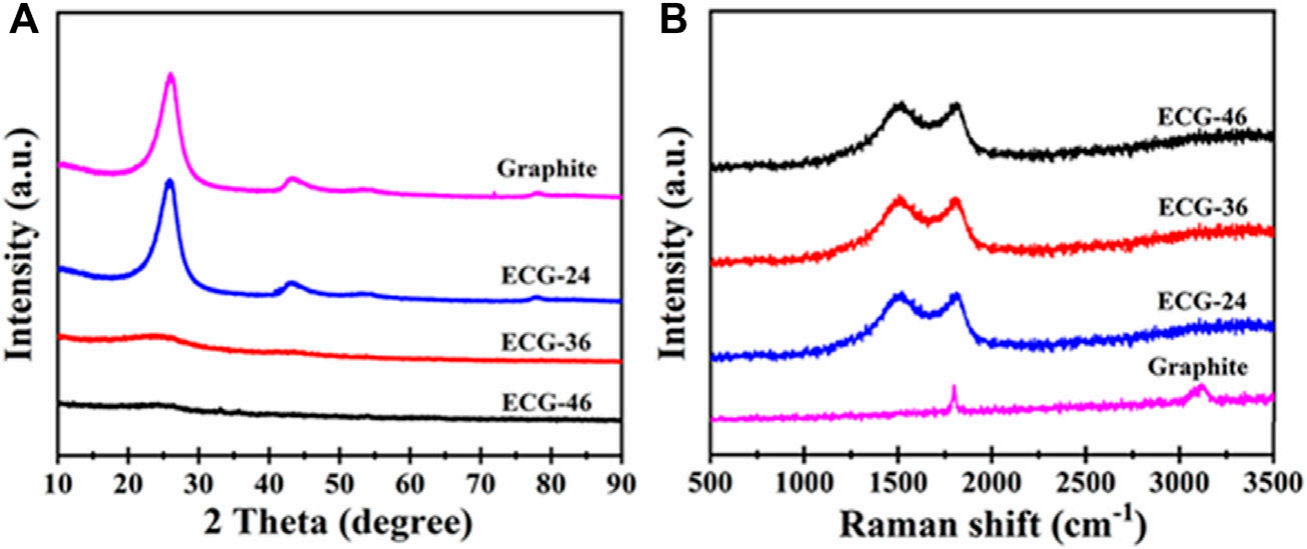
**(A)** Diffraction patterns and **(B)** Raman spectra of pristine graphite and ECG samples.

Figure 3A shows the structural differences in the X-ray diffraction patterns of ECG-46 and ECG-36, ECG-24, and pristine graphite to provide more insight into the growth process of the ECGs as a result of varying milling times. When compared to graphite and ECG-24, the sharpness and negative shift in ECG-46 and ECG-36 were caused by COOH at the edges of the carbon matrix, which altered the spacing of the interior layers due to the rising change in ball milling time. There was only a broad diffraction peak at 26.6° for ECG-46 and ECG-36, which is typical of the (002) lattice plane of disordered carbon due to the carboxylate modification. The lowering of the peak of ECG-46 different from ECG-36 suggests the strong carboxylate modification effect. On the other hand, graphite and ECG-24 displayed obvious diffraction peaks at 2θ positions of 26.3 and 45.5°, which are characteristic of (002) and (100) lattice planes correspondingly attributed to graphitic carbon layers and hexagonal graphite (Hamouda et al., 2022). Furthermore, Raman spectra were taken to examine the modification effect. The ECG-46, ECG-36, and ECG-24 results showed a typically wide range of graphitic carbon at 1592 cm^−1^ (band G) and crystalline graphite sp^2^ carbon at 1343 cm^−1^ (band D). On the other hand, pristine graphite demonstrated a graphitic carbon band at 1592 cm^−1^ (band G) and an extra two-dimensional band at 2901 cm^−1^. The graphite band shifted more positively than graphite due to edge carboxylation, considering the larger intensity ratio of ECG-46 (0.6) against graphite (0.2) as observed in Figure 3B.

**FIGURE 4:**
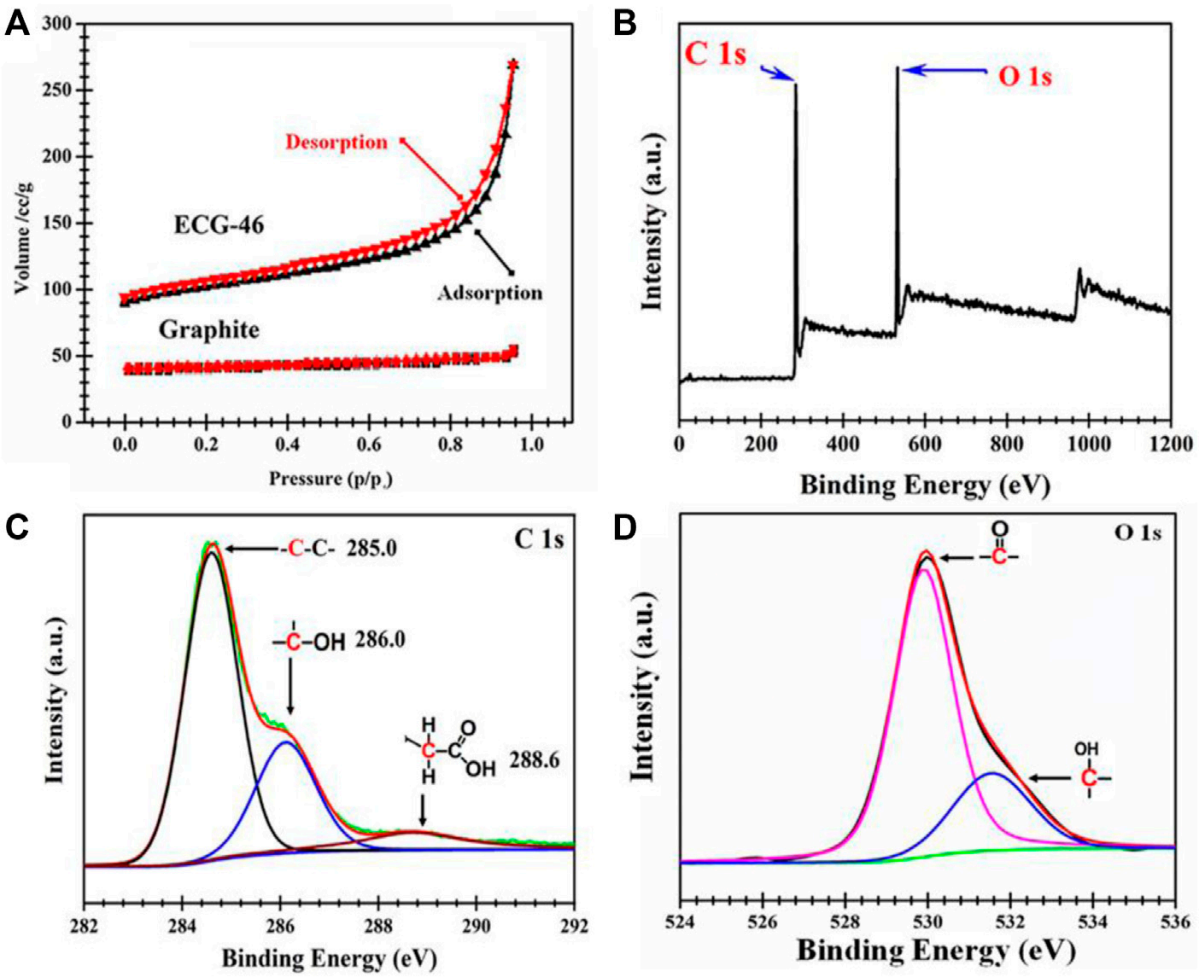
**(A)** Nitrogen sorption isotherms; XPS spectra of the ECG-46 nanosheet, **(B)** Survey scan **(C)** C 1s core level spectrum and **(D)** O 1s level spectrum.

Figure 4A shows the N_2_ sorption isotherms of ECG-46 and pristine graphite. In contrast to pure graphite, which has a type I isotherm, the ECG-46 sample has a type IV isotherm with steep adsorption at high relative pressure (P/P_0_) = 0.96, indicating large mesoporous surface area in its structure. To have a better understanding of the modification effect, ECG-46 was subjected to an XPS analysis. The XPS survey scan spectra revealed two prominent peaks for C 1s and O 1s at 284.3 eV and 531.7 eV respectively, indicating a higher number of oxidations (Figure 4B). Interestingly, the resolution spectrum of C 1s of ECG-46 sample (Figure 4C) displayed the primary peaks assigned to C=O of carbonyl, C-O-C, and C-C/C=C, C-O of (hydroxyl), and for carboxylic acid O-C=O, however, O 1s core level spectrum were attributed to C=O, C-O, and OH, bonds Figure 4D.

### Electrochemical analysis results

**FIGURE 5:**
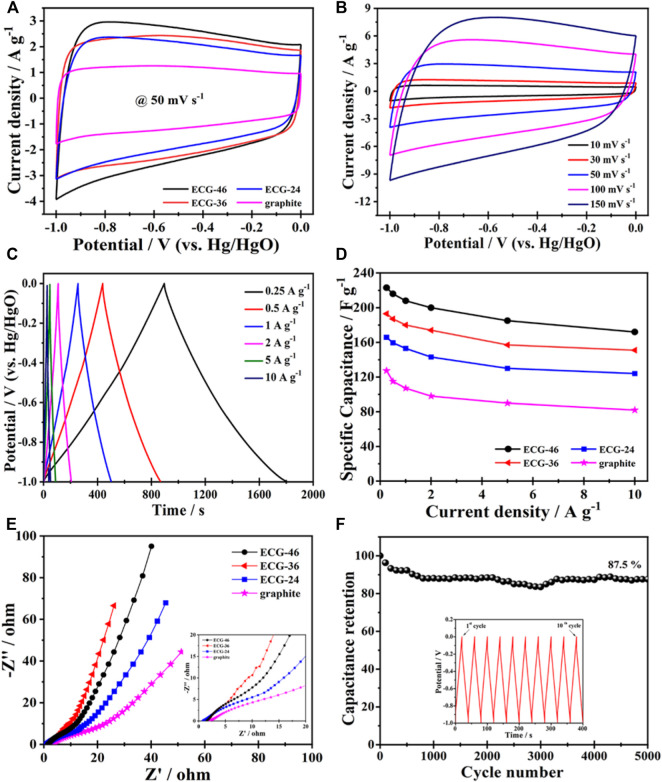
The CV curves of **(A)** ECG-46, ECG-36, ECG-24 and graphite at 50 mV s^−1^ and **(B)** ECG-46 with respect to scanning rate; **(C)** The GCD curves with respect to current densities of EGC-46; **(D)** The specific capacitances of EGC-46, EGC-36, EGC-24 and pristine graphite; **(E)** Nyquist plot of the ECG-46, ECG-36, ECG-24 and graphite electrodes; **(F)** the cycling stability of the ECG-46 in 2 M KOH electrolyte (Inset presents the GCD curves of 1st to 10th cycles).

The electrochemical performance of pure graphite and the three ECG electrodes were first investigated in a three-electrode system. Figure 5A shows the cyclic voltammetry (CV) curves of ECG-46, ECG-36, ECG-24, and graphite at a scan rate of 50 mV s^−1^. All the ECGs have suitable rectangular shapes in their CV curves, signifying a quick electrochemical response and good power properties. However, when graphite was utilized without ball milling, the CV curve showed a short irregular rectangular curve, demonstrating poor capacitance (Keithley et al., 2011). The CV curve of the best sample, ECG-46, was then tested at various scan rates (Figure 5B). The CV curves revealed excellent rectangular shapes when the scan rate was increased to 100 mV s^−1^, showing that the ECG-46 had rapid charge transfer and good rate capacity. The symmetrical galvanostatic charge-discharge (GCD) curves of the ECG-46 at different current densities are shown in Figure 5C and indicate strong electrochemical reversibility. Furthermore, the formula in Eq. 1 was used to calculate the specific capacitances for the three-electrode system from GCD curves. Figure 5D shows that of the samples examined, ECG-46 had the highest specific capacitance of 223 F g^−1^ at 0.25 A g^−1^, followed by ECG-36, ECG-24, and graphite with 193, 165, and 127 F g^−1^, respectively as well to these results, it was also compared with others electrodes materials (see Table 1 for more details). The integrated hierarchical nano-framework structure gave the ECG-46 nanosheets a substantial surface area and structural stability. The Nyquist plot (Figure 5E) displays the electrochemical impedance spectra of the ECG electrodes before the 5000 cycle test while the inset of Figure 5E showed a small semicircle in the high-frequency zone and a sloping line in the low-frequency region, suggesting their low charge transference resistance and quick electrolyte diffusion rate. The ECG-46 exhibited a low equivalent series resistance (Rs) of 1.56Ω, indicating that the electroactive material resistance, the electrolyte’s ionic resistance, and the electrolyte’s interface contact resistance were low. The charge transference resistance (Rct) at 10.94 indicates a common charge transference resistance due to the Faradaic reaction and the best capacitance performances with low diffusion resistance (Ahmad et al., 2020; El-Hout et al., 2021; Tu et al., 2021). In addition, the strategy of ball milling graphite is a practical approach to improve their electrochemical characteristics. Because a higher specific surface area allows for a better electrochemical property, this is the case. In the 2 M KOH electrolyte and charge-discharge of 5 A g^−1^, Figure 5F showed the cycling stability of the ECG-46 (three-electrode system). Clearly, after charge/discharge stability test of 5000 cycles, the cell retained 87.5% of its original value, demonstrating outstanding cycling stability. Inset Figure 5F depicts the first ten (1st to 10th) GCD cycles of ECG-46, which demonstrates regular charge/discharge in the GCD curves. The preparation method of the ECGs can generate a high number of channels for effective electrolyte diffusion and enhance the material’s conductivity and specific capacitance (Srivastav, Paliwal, and Meher, 2022), which is responsible for the exceptional electrochemical properties and performance.

**TABLE 1:** Comparison of specific capacitances of literature results obtained from materials of carbon-based precursors with this work.

Carbon precursor	Electrolytes	Specific capacitance	Current density	References
Pine cone	1 M Na_2_SO_4_	137 F g^−1^	0.1 A g^−1^	Bello et al. (2016)
Corn stalk core	3 M KOH	140 F g^−1^	1 A g^−1^	Yu et al. (2018)
PRPC-1K	2 M KOH	170 F g^−1^	0.5 A g^−1^	Peng et al. (2019)
Garlic peels	4 M KOH	174 F g^−1^	0.1 A g^−1^	Bhat et al. (2020)
HBFC-1	2 M KOH	194.5 F g^−1^	0.5 A g^−1^	Hamouda et al. (2021)
ZLPC	2 M KOH	196 F g^−1^	0.5 A g^−1^	Xu Y. et al. (2020)
**ECG-46**	**2 M KOH**	**216 F g** ^**−1**^	**0.5 A g** ^**−1**^	**This work**

The bold values are values obtained from this work.

**FIGURE 6:**
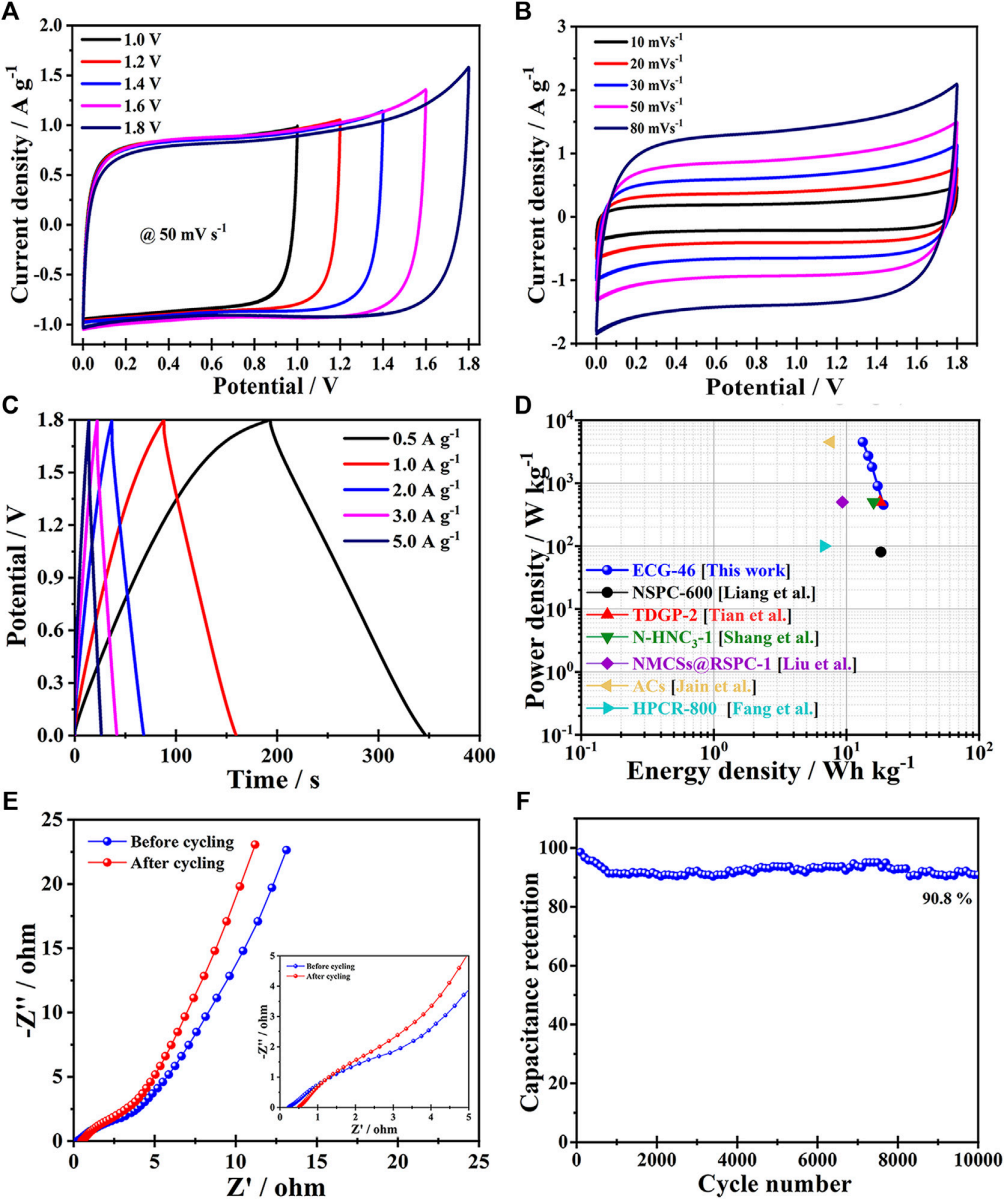
**(A)** CV curves of the ECG-46 symmetric two-electrode cell (50 mV s^−1^) in 0.5 M Na2SO4 aqueous electrolytes at various voltage windows; **(B)** Different scan rates of symmetric supercapacitor CV curves; **(C)** GCD curves at various current densities for symmetric supercapacitors; **(D)** Ragone plots of ECG-46 vs. other carbon-based supercapacitors; **(E)** Nyquist graphs of ECG-46 supercapacitor; **(F)** Cycling stability of the ECG-46 symmetric supercapacitor.

To learn more about the electrochemical performance of the ECG-46 electrode-based supercapacitor in a two-electrode system, an ECG-46 symmetric supercapacitor (ECG-46/ECG-46 SSC) was assembled and tested for cyclic voltammetry, galvanostatic charge/discharge, and electrochemical impedance spectroscopy in a 0.5M Na_2_SO_4_ electrolyte (EIS). The CV of the ECG-46/ECG-46 SSC maintained a nearly rectangular shape at each voltage of operation (Figure 6A), showing a better capacitive behaviour. As seen in the figure for the test conducted at 1.8 eV, the continuous increase in voltage 1.0 V–1.6 V changed the perfect rectangular shape to a sharp increase, indicating the most ideal behavior with large amplitude. As a result, the potential power of 1.8 eV was chosen to test for CV of ECG-46//ECG-46 SSC at different scan rates (10–80 mV s^−1^) and the curves are displayed in Figure 6B. The CV of the ECG-46/ECG-46 SSC maintained a nearly rectangular shape at each voltage of operation (Figure 6A), showing perfect capacitive behavior. It was observed that the shape of the CV curves did not change significantly even at the highest scan rate. This indicates good rate ability and fast ion transfer. Figure 6C shows GCD curves of the ECG-46/ECG-46 SSC at 1.8 V in the range of 0.5–5 A g^−1^. The specific capacities were 43, 40, 35, 33, and 30 F g^−1^ for current densities of 0.5, 1, 2, 3, and 5 A g^−1^, respectively, demonstrating that the discharge curves is very symmetric with their parallel counterparts and that the charge and discharge curves are symmetric with time change. As a result, the ECG-46/ECG-46 SSC exhibits strong electrochemical reversibility.

The fundamental purpose of Ragone plots is to relate energy and power density in order to positively affect high-performance supercapacitors. When the power density was 450 W kg^−1^, the highest energy density was 19.2 Wh kg^−1^, which declined to 13.67 W h kg^−1^ when the power density was reduced. When the power density is increased to 4500 W kg^−1^, the ECG-46//ECG-46 SSC has a higher energy density than previously reported SSC devices such as the NSPC-600 SSC (18.20 Wh kg^−1^), TDGP-2 SSC (17.90 Wh kg^−1^) (Tian et al., 2019), N-HNC_3_-1 SSC (15.99 Wh kg^−1^) (Shang et al., 2020), NMCSs@RSPC-1 SSC (9.31 Wh kg^−1^) (Liu et al., 2018), ACs SSC (7.6 Wh kg^−1^) (Jain et al., 2015), and HPCR-800 SSC (6.77 Wh kg^−1^) (Fang et al., 2019) as shown in Figure 6D, and Table 2. The previously selected voltage range (1.8 V) increases the power density of the supercapacitor according to the equation E=1/2CV^2^.

**TABLE 2:** Comparative electrochemical performance of ECG-46//ECG-46 symmetric device with previously reported supercapacitors.

Symmetric device	Energy density	Power density	Cycling stability	References
NSPC-600//NSPC-600	18.2 Wh L^−1^	80.4 W L^−1^	91.2% after 10,000 cycles	Liang et al. (2021)
TDGP-2//TDGP-2	17.9 Wh kg^−1^	500 W kg^−1^	89.0% after 5,000 cycles	Tian et al. (2019)
N-HNC_3_-1//N-HNC_3_-1	15.99 Wh kg^−1^	500 W kg^−1^	95.74% after 10,000 cycles	Shang et al. (2020)
HPC-700//HPC-700	14.4 Wh kg^−1^	225 W kg^−1^	93.0% after 15,000 cycles	Zhao et al. (2020)
SSC1.0//SSC1.0	9.77 Wh kg^−1^	225.35 W kg^−1^	92.0% after 5,000 cycles	Ma et al. (2016)
NMCSs@RSPC-1 SSC	9.31 Wh kg^−1^	500 W kg^−1^	96.0% after 10,000 cycles	Liu et al. (2018)
ACs//ACs	7.60 Wh kg^−1^	4.5 kW kg^−1^	90.0% after 2,000 cycles	Jain et al. (2015)
HPCR-800//HPCR-800	6.77 Wh kg^−1^	100 W kg^−1^	81.0% after 10,000 cycles	Fang et al. (2019)
**ECG-46//ECG-46**	**19.2 Wh kg** ^**−1**^	**450 W kg** ^**−1**^	**90.8% after 10,000 cycles**	**[This Work]**

The bold values are values obtained from this work.

The Nyquist plot of the ECG-46/ECG-46 SSC, as shown in Figure 6E, offers a semicircular line in the high frequency range, most likely due to interfacial charge resistance, while the vertical line displays excellent capacitive behaviour at low frequencies. The frequency level, which represents the frequency band with the lowest Warburg resistance, is linked to rapid ion transport between the electrode and the electrolyte. The resistance of ECG-46/ECG-46 SSC after charge-discharge cycling was found to be higher than that of ASC before cycling (Rs = 0.23, Rct = 2.91), indicating that ECG-46//ECG-46 SSC has good charge transfer efficiency, fast ion diffusion between electrode and electrolyte, and excellent electrochemical stability. After 10,000 cycles, the capacitance retention rate of the ECG-46//ECG-46 supercapacitor at 2 A g^−1^ was as high as 90.8% (as shown in Figure 6F), demonstrating that the ECG-46//ECG-46 SSC exhibits good cycling stability. The result could be due to the porous interconnected carbon nanosheet structure as well as the rise in oxygen content generated by the addition of dry ice to the graphene material, implying that the supercapacitor has strong capacitive capabilities.

## Conclusion

In conclusion, we have demonstrated the use of a series of porous carboxylated graphitic material as active electrode for high-performance supercapacitor that differs from pristine graphite. Due to its interconnected micro-mesoporous carbon network. The ECG-46 electrode in a two-electrode system has a high energy density of 19.20 W h kg^−1^ at 450.00 W kg^−1^ power density, allowing for rapid ion transport, ion buffering, and ion storage while also ensuring that the porous surfaces are well utilized. The large micro-mesopore structure of ECG-46, in particular, is necessary for fast charge-discharge at high current densities, improved rate capability performance, storing static charges, and enhancing electrolyte transport and diffusion. Micropores have large specific surface areas for storing static charges, whereas interconnected mesopores aid in electrolyte transport and diffusion. This study emphasizes the importance of carboxylate ion functions in supercapacitor design and suggests to a new route for active electrode design. A promising strategy for tuning the performance of graphitic materials would be to precisely control balling time and the deposition of active oxygen functions.

## Data Availability

The original contributions presented in the study are included in the article/supplementary material, further inquiries can be directed to the corresponding author.
